# The Impact of the COVID-19 Pandemic on Gastrointestinal Endoscopy Activity in a Tertiary Care Center from Northeastern Romania

**DOI:** 10.3390/healthcare9010100

**Published:** 2021-01-19

**Authors:** Stefan Chiriac, Carol Stanciu, Camelia Cojocariu, Catalin Sfarti, Ana-Maria Singeap, Irina Girleanu, Tudor Cuciureanu, Laura Huiban, Diana David, Sebastian Zenovia, Robert Nastasa, Gheorghe G. Balan, Anca Trifan

**Affiliations:** 1 Medicale I Department, “Grigore T. Popa” University of Medicine and Pharmacy, 700115 Iasi, Romania; stefannchiriac@yahoo.com (S.C.); cvsfarti@gmail.com (C.S.); anamaria.singeap@yahoo.com (A.-M.S.); gilda_iri25@yahoo.com (I.G.); drcuciureanutudor@gmail.com (T.C.); huiban.laura@yahoo.com (L.H.); sebastianzenovia20@gmail.com (S.Z.); nastasa.robert@yahoo.com (R.N.); balan.gheo@yahoo.com (G.G.B.); ancatrifan@yahoo.com (A.T.); 2 Institute of Gastroenterology and Hepatology, “St. Spiridon” Emergency Hospital, 700111 Iasi, Romania; stanciucarol@yahoo.com (C.S.); dianaelena.dav@gmail.com (D.D.)

**Keywords:** coronavirus disease, gastrointestinal endoscopy, colonoscopy, ERCP, gastrointestinal cancer

## Abstract

Background: The outbreak of the coronavirus disease 2019 (COVID-19) has led to significant changes in endoscopy units worldwide, with potential impact on patients’ welfare as well as on endoscopy training. We aimed to assess the real-life impact of COVID-19 on the endoscopy unit in a tertiary care center from Romania. Methods: A 6.5-month period during the COVID-19 pandemic was compared to a similar period from 2019. Results: A 6.2-fold decrease of endoscopic procedures was noted. Colonoscopies were reduced from 916 to 42, *p* < 0.001; flexible sigmoidoscopies from 189 to 14, *p* = 0.009; upper gastrointestinal (GI) endoscopies from 2269 to 401, *p* = 0.006; and ERCP from 234 to 125, *p* < 0.001. The percentage of emergency procedures increased (38.8% vs. 26.2%, *p* < 0.001), as well as the rate of endoscopies performed for upper GI bleeding (42.5% vs. 24.4%, respectively, *p* < 0.001). The detection of cancers was considerably reduced (57 compared to 249, *p* = 0.001). There were fewer complications and higher success rates (7.6% vs. 19.2%, *p* < 0.001, and 94.2% vs. 90.7%, respectively). Fellows participation was also reduced from 90% to 40.9% (*p* < 0.001). Conclusions: The COVID-19 pandemic has significantly altered the workflow of the endoscopy unit, lowering the number of procedures performed and potentially compromising the early detection of cancers.

## 1. Introduction

Since the first detection of the SARS CoV-2 virus in patients with lower respiratory tract infection of unknown etiology in December 2019 in the Wuhan, Hubei Province, China [1], over 29 million patients have been diagnosed, resulting in over 900,000 deaths worldwide [2]. In February 2020, the World Health Organization (WHO, Geneva, Switzerland) established the name of the disease caused by the SARS CoV-2 infection as the coronavirus disease 2019 (COVID-19), in March 2020, declared it a pandemic [3]. The gastrointestinal (GI) endoscopy departments worldwide were severely affected, and elective procedures were postponed in most centers, leading to an unprecedented decrease of the endoscopic workload globally [4,5,6,7]. The significant impact of COVID-19 has been partly attributed to the high contagious potential of the SARS CoV-2 virus as well as the long incubation time. The main transmission pathways are through exposure to air droplets or through direct contact [8]. However, alternative transmission has been shown via small airborne particles [9] as well as through the fecal-oral pathway [10]. The potential for transmission is high during aerosol-generating procedures such as GI endoscopy. Thus, the prioritization of these procedures has been advocated by many international endoscopy organizations such as World Endoscopy Organization (WEO, Munich, Germany) [11], European Society of Gastrointestinal Endoscopy (ESGE, Munich, Germany), European Society of Gastroenterology and Endoscopy Nurses and Associates (ESGENA, Munich, Germany) [12], Asian Pacific Society for Digestive Endoscopy (APSDE, Japan) [13], American Society for Gastrointestinal Endoscopy (ASGE, Downers Grove, USA) [8], and American Gastroenterological Association (AGA, Bethesda, USA) [14]. These societies have issued recommendations such as the mandatory use of personal protective equipment (PPE) and the requirement for negative pressure rooms, and also suggest viral testing based on polymerase chain reaction (PCR) where available, thus substantially increasing the costs of GI endoscopy and potentially leading to an even further decrease of the number of procedures [15]. They have stated that nonurgent procedures should be deferred, and only emergency GI endoscopy should be performed in order to prevent cross contamination and to reduce the risk for the endoscopy unit personnel. Moreover, the guidelines suggest reducing the onsite endoscopy staff and maintaining only the personnel essential for the procedure, creating special circuits for patients, monitoring temperature of patients and staff, and applying questionnaires for symptoms or previous exposure. All of these measures, combined with the patients’ fear of contracting the SARS-CoV2 infection from hospital milieu, have resulted in the dramatic general decrease of GI endoscopies worldwide [4,5,6,7]. The impact of these measures is currently being evaluated, and the available data indicate a significant decrease in the GI cancer detection rate [5] and a reduction in trainees’ opportunities of participating in GI endoscopy [16]. The measures, mainly directed toward the protection of personnel, are based on data suggesting a high risk of transmission of the SARS CoV-2 virus in the endoscopy unit [8]. However, recent data has suggested that appropriate use of PPE and recommended measures can significantly reduce this risk [17].

We aimed to assess the impact of the COVID-19 pandemic on GI endoscopy in a tertiary care center in northeastern Romania concerning the number of procedures performed, indications, complications, and results as well as trainee involvement.

## 2. Materials and Methods

The database containing GI endoscopies performed in the “St. Spiridon” Emergency Hospital, Institute of Gastroenterology and Hepatology, Iasi, Romania was analyzed, and information was retrieved in a confidential manner. Two time periods were considered for comparison, namely 1st of March–15 September 2020 during the COVID-19 pandemic, and a similar period between 1st of March–15 September 2019. Patients’ age and sex, as well as the time and type of the procedure were recorded. The indication, urgency, endoscopic diagnosis, procedures performed, success rates, and complications were also noted, as well as the participation of fellows. Incomplete records were excluded from the study.

The study performed was a service evaluation. Thus, there was no requirement for ethical approval.

### 2.1. Internal Protocol

The endoscopic procedures were performed in accordance with the established COVID-19 internal protocol, which stated that no ambulatory procedures could be carried out and that endoscopy should be performed only in hospitalized patients. The decision for hospitalization was at the discretion of the on-call doctor via the hospital emergency department. The indications for endoscopy were restricted to either GI bleeding or to cases considered at high risk for cancer.

The hospital followed the general international recommendations concerning the reduction of nonessential personnel in the endoscopy room, PPE use, temperature monitoring, and special circuits, as well as personal and patient questionnaires assessing the presence of symptoms of the COVID-19 disease. All patients were tested for SARS CoV2 infection by PCR and endoscopy was deferred until a negative test was available, with the exception of emergency procedures.

Trainees were no longer included when emergency procedures were performed in order to minimize the risk of contamination and to decrease the need for PPE. The decision to include fellows in nonurgent procedures was taken on a case-by-case basis by the endoscopist.

### 2.2. Statistical Analysis

IBM Statistical Package for Social Sciences (SPSS, Armonk, NY, USA) version 22.0 was used for the statistical analysis. The Kolmogorov-Smirnoff test was used to assess the distribution of the continuous variable (age), which was expressed as median (interquartile range (IQR)) as it presented a nonparametric distribution. Categorical variables were expressed as frequency and percentage. Absolute numbers of endoscopic procedures and percentage reductions were calculated. Chi-square or Fischer’s test was used for the analysis of categorical variables accordingly. Statistical significance was considered for a *p*-value of less than 0.05.

## 3. Results

### 3.1. General Findings

**Figure 1:**
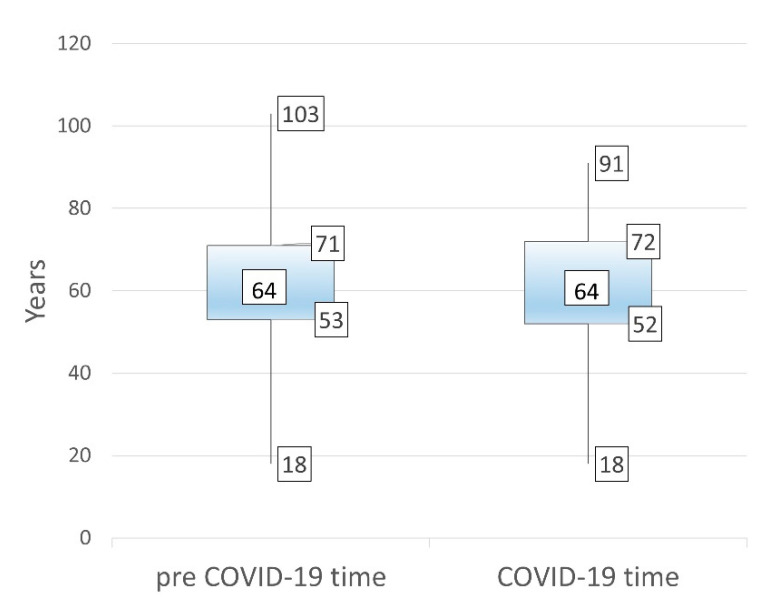
Median values of the age of the patients before and during the COVID-19 period.

There was a general 6.2-fold decrease in the total number of procedures as a result of the COVID-19 restrictive recommendations. During the pre-COVID-19 period, 3608 endoscopic procedures were carried out, with a mean of 138 per week. During the COVID-19 period, only 582 procedures were performed, approximatively 22 procedures per week. The percentage of male patients and emergency endoscopies increased during the COVID-19 period. The general characteristics of endoscopies before and during COVID-19 period are described in Table 1 and Figure 1.

**Figure 2:**
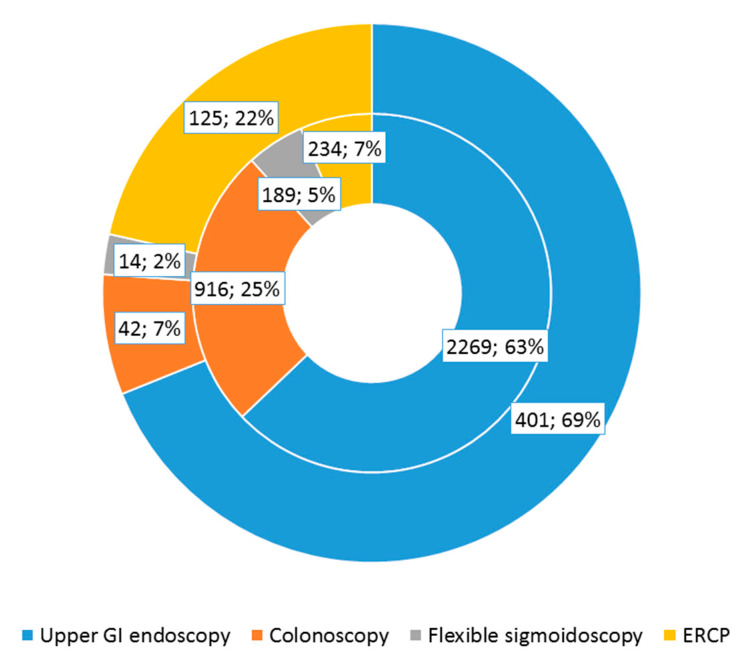
Impact of COVID-19 on the numbers of endoscopic procedures.

Although the absolute number of procedures decreased, a significant increase in the relative percentage of upper GI endoscopy and endoscopic retrograde cholangiopancreatography (ERCP) was noted, together with a significant decrease in the percentage of colonoscopies and flexible sigmoidoscopies as a result of COVID-19 imposed restrictions. The most significant reduction was found in colonoscopy, from 916 to 42 procedures, *p* < 0.001; followed by flexible sigmoidoscopy, from 189 to 14 procedures, *p* = 0.009; upper gastrointestinal endoscopy, from 2269 to 401 procedures, *p* = 0.006; and ERCP, from 234 to 125 procedures, *p* < 0.001 (Figure 2).

### 3.2. Training Analysis

Compared to the pre-COVID-19 period, during the COVID-19 period, a significant reduction in fellows’ involvement in endoscopy procedures was noted (40.9% vs. 90%, respectively, *p* < 0.001).

### 3.3. Upper and Lower GI Endoscopy Analysis

The detailed results of the analysis of upper and lower endoscopic procedures are presented in Table 2. Concerning the indications for upper and lower GI endoscopy during the COVID-19 period compared to the pre-COVID-19 period, there was a significant increase in the percent of endoscopies performed for upper GI bleeding (42.5% vs. 24.4%, respectively, *p* < 0.001), but a reduction in colonoscopies performed for lower GI bleeding (4.4% vs. 8.6%, respectively, *p* = 0.002). Moreover, endoscopies performed for several indications such as chronic abdominal pain, weight loss, control of gastric ulcer, polyps, or cancer were significantly reduced, whereas procedures indicated for anemia, change in the frequency of stools, vomiting, dysphagia, or screening for esophageal varices were not impacted by the COVID-19 restrictions. The changes in the frequency of endoscopic diagnostics were in accordance with the more restrictive indications. Thus, a significant increase in the detection of GI neoplasia was noted in the COVID-19 period (12.5% vs. 7.4%, respectively, *p* < 0.001). Moreover, gastric and duodenal ulcers were more frequently diagnosed during the pandemic compared to the pre-COVID period, but significant differences were only noted in the case of Forrest III ulcers (8.8% vs. 5.5%, respectively, *p* = 0.002). The diagnostic frequency of other conditions, such as gastritis, esophagitis, achalasia, esophageal benign stenosis, inflammatory bowel disease, diverticular disease, or hemorrhoids, was not significantly modified. However, normal findings in endoscopy were significantly reduced (0.2% vs. 2%, *p* = 0.006). Concerning endoscopic interventions performed before and during the COVID-19 period, there was a general increase in variceal band ligation, bougie and balloon dilation of esophageal strictures, esophageal stent placement for neoplasia, and percutaneous endoscopic gastrostomy (PEG), but with no notable statistical significance with the exception of gastric or duodenal ulcer hemostasis (4.5% vs. 2.8%, respectively, *p* = 0.039). The COVID-19 pandemic determined an important decrease of polypectomies and endoscopic mucosal resections (EMR)s (0.2% vs. 5%, respectively, *p* < 0.001). A significant reduction of early complications was noted (7.6% vs. 19.2%, respectively *p* < 0.001). There were seven cases of delayed complications during the pre-COVID period but none during the COVID-19 period. An increased rate of therapeutic success was noted during the pandemic (94.2% vs. 90.7%, respectively, *p* = 0.01) compared to the pre-COVID period (Table 2).

### 3.4. ERCP Analysis

The change in ERCP indications induced by the COVID-19 restrictions was less dramatic than in the case of upper and lower GI endoscopies. Some differences, although without statistical relevance, were expressed as a relative increase in the percent of procedures carried out for neoplasia, such as pancreatic cancer, cholangiocarcinoma, lymph node, or hepatic metastasis. A reduction of ERCPs performed was found for common bile duct (CBD) stone extraction and postoperative biliary lesions. As a result of the increase in the percentage of difficult cases, the rate of success diminished during the COVID-19 period compared to pre-COVID period (76.3% vs. 87.7%, respectively, *p* = 0.006). However, there was a relative decrease in complications, reflected notably in a reduced immediate bleeding rate (7.3% vs. 11.6%, respectively, *p* = 0.196) (Table 3).

## 4. Discussion

We analyzed the impact of the COVID-19 pandemic on the number, type of endoscopic procedures, and outcomes in a tertiary care unit from northeastern Romania. The information collected from our database indicated that there was a significant general decrease of the number of endoscopic procedures performed during the pandemic. This reduction in absolute numbers has also been reported in other regions, such as the United States of America [18], United Kingdom (UK) [5], the Netherlands [19], and China [20]. The most dramatic impact was the reduction in the number of colonoscopies and flexible sigmoidoscopies performed during the pandemic, followed by upper GI endoscopies. A lesser reduction was found in ERCPs. Similar results were reported by Rutter et al. in a national analysis of the impact of the COVID-19 pandemic on the endoscopic activity in the UK. The authors reported that colonoscopies, flexible sigmoidoscopies, and gastroscopies were reduced by 90%, 91%, and 86% respectively, but that ERCPs were reduced by only 44% [5]. In our center, we found that although the absolute numbers of endoscopic procedures were decreased, there was an increase in the percent of upper GI endoscopies as well as ERCPs, whereas the percent of colonoscopies and flexible sigmoidoscopies decreased. These changes occurred as a result of the restrictive indications adopted by our institution, limiting the procedures to either GI bleeding or to cases considered at high risk for cancer. Thus, indications such as chronic abdominal pain, weight loss, control of gastric ulcer, post polypectomy control, or postoperative cancer surveillance were considerably reduced. Our results are in accordance with data from a multicenter study carried out in Italy that indicated that most endoscopy units also limited their indications to emergency procedures or to cases presenting high risk for digestive cancer. Thus, the Italian endoscopy departments reported a subsequent total reduction in endoscopic procedures varying from no reduction to 100% [7]. In our institution, we found a significant relative increase of endoscopies performed in emergency setting. As a consequence, the upper and lower GI endoscopy findings were also different when compared to the pre-COVID-19 period. The considerable reduction of activity of the endoscopy department had a significant effect on the detection of cancers. The absolute number of cancers detected as a result of the COVID-19 pandemic decreased dramatically. This decrease is in accordance with the worldwide trend induced by the impact on cancer screening programs as well as by the more restrictive indications for endoscopic procedures [5,7]. Even though we found an absolute significant decrease in the detected GI neoplasia, the relative percentage of GI neoplasia diagnosed during the pandemic was considerably higher. Similar findings have also been reported in the UK where a general reduction of all digestive cancers was noted, with an over three-fold increase in the cancer detection rate as a result of more restrictive endoscopy indications [5]. In the Netherlands, a significant decrease in the detection of cancers was also observed as a result of the pandemic [19].

Moreover, the rates of gastric and duodenal bleeding ulcers as well as esophageal varix diagnostic were increased. As expected, the percent of GI polyps and normal endoscopic findings was considerably diminished. Concerning the endoscopic interventions, we noted an increase in the percentage of hemostasis, both for variceal and nonvariceal upper GI bleeding, but a significant decrease in polypectomy and EMR, in accordance with the considerable reduction in colonoscopies and flexible sigmoidoscopies in favor of upper digestive endoscopies. Surprisingly, the success rates were higher and there were less complications related to endoscopy during the COVID-19 pandemic. These findings could indicate a higher concern of the endoscopy personnel aiming to reduce the need for subsequent endoscopic procedures, either because of failure of the intervention or because of the development of complications.

The number of ERCPs was reduced by almost a half during the COVID-19 period. However, this decrease was less substantial when compared to the other GI procedures. Concerning the indications, a shift toward palliative treatment of cholangiocarcinoma and pancreatic cancer was noted, as well as a relative reduction of procedures carried out for common bile duct stone extraction. As a result, the success rate of ERCPs during the pandemic was relatively lower, although without statistical significance. Other studies throughout the world have reported similar results concerning ERCPs, presenting a global reduction of procedures but to a lesser extent when compared to other upper or lower GI endoscopies [5,7].

The fellows’ involvement in the endoscopy department was considerably reduced as a result of the need for judicious use of PPE and of the recommendations imposing the minimization of personnel exposure during endoscopy. Similar results have been reported throughout the world. Forbes N et al., in a study comprising 73 institutions from North America and Canada, found that 49% of the centers included had eliminated endoscopic training altogether, and 45% had completely stopped interventional endoscopy training programs [18]. A service evaluation of the endoscopy activity in the UK found a 93% reduction in the rate of endoscopic procedures carried out by fellows [5]. An international study including 770 participants from 63 countries and 6 continents showed a reduction of trainee involvement in endoscopic procedures in 93.8% of cases. The participants evaluated that the decision to exclude the trainees from endoscopic procedures was taken in 79.9% of cases, but that a lack of cases or a shortage of PPE was noted in 58.3% and in 28.8% of cases, respectively [16].

Most endoscopy units throughout the world are currently working to resume the elective GI endoscopies that have been delayed during the COVID-19 imposed restrictions. This is a difficult but necessary process as the worldwide dramatic reduction in the number of procedures affects patients at risk by potentially delaying the diagnosis of cancer [21]. Although the global health crisis is far from over, the need to gradually restart the endoscopy units has led several international societies to publish recommendations regarding the resumption of elective endoscopic procedures [22,23]. These recommendations include the thorough preparation of patients, health care providers, equipment, and infrastructure in order to minimize the risk of exposure and assure a constant on-duty endoscopy personnel in the case that part of the staff becomes infected and needs to be quarantined [8,24]. However, these measures could be difficult to implement in certain settings where the available infrastructure is not adequate or where there are shortages of available PPE. Clearly, the new road ahead is challenging, and the recommendations need to be tailored to the existing local possibilities in order to balance the risk of COVID-19 dissemination with the risk associated with the lack of timely endoscopic interventions [25]. In our institution the recommendations of PPE use, as well as the introduction of specific circuits and patient hygiene, has been implemented since the beginning of the pandemic. PCR viral testing was offered to all of the patients before endoscopy. These measures, combined with the judicious use of the PPE, led to the reduced number of nonurgent procedures performed. However, no urgent endoscopy was postponed. The endoscopists prioritized the procedures in accordance with the individual risks of the patients, and sometimes even performed endoscopies in disregard of their own personal safety when deferring the procedure would have put the patient in danger. Although several members of the endoscopy staff have contracted the COVID-19 disease, it was difficult to firmly establish a direct link between the infected patients and these cases as community transmission was also possible. A more in-depth analysis concerning the epidemiological investigation regarding the cases is necessary in order to be able to confidently present those results. In the light of the ongoing vaccination program, the resumption of elective endoscopy should be prioritized. The prioritization of cases should be continued, and previously postponed endoscopies should be gradually performed. Efforts should be made to achieve a good communication with the patients. This is particularly important in order to assure them that measures are being taken to assure their safety during and after the procedure.

The COVID-19 pandemic is the fifth pandemic since the Spanish Flu from 1918 and it will most probably not be the last. Thus, the experience that we gained during this difficult time will provide the basis for future action against other potential pandemics. The judicious use of PPE and the detailed instructions for patient and personnel hygiene, as well as the development of specific circuits and the more rigorous implementation of good practice protocols, are here to stay and represent a solid foundation in the fight against future pandemics.

Our study is the first to assess the impact of COVID-19 on the endoscopy practice in Romania. However, it presents some limitations. First, the study was based on a single-center analysis. Also, the database did not contain information on the rate of ERCP-related delayed complications. Thus, it could not be evaluated.

## 5. Conclusions

The COVID-19 pandemic has had a profound impact on the endoscopy department, characterized by a consistent reduction of upper and lower GI procedures, as well as a more modest reduction of ERCPs. The overall cancer detection rate was significantly reduced, indicating a risk for missing the opportunity of potentially curative treatment due to the pandemic. Endoscopic procedures were mostly carried out in emergency setting, but therapeutic procedures were associated with less complications and higher success rates. Only in time we would be fully able to understand the real impact of the COVID-19 pandemic on the well-being of our patients. However, the results suggest that this impact would result in an increased number of advanced and potentially unresectable cancers, as well as a high risk of morbidity and mortality. Thus, efforts should be made in order to safely reopen the endoscopy departments.

## Figures and Tables

**Table 1:** General characteristics of endoscopic procedures performed before and during the COVID-19 period.

Characteristic	Pre COVID-19 Time 3608	COVID-19 Time 582	*p*
Sex, women:men, *n* (%)	1620 (44.9):1987 (55.1)	235 (40.4):347 (59.6)	0.041
Emergency upper/lower GI endoscopy, *n* (%)	947 (26.2)	226 (38.8)	<0.001
Fellow involvement, *n* (%)	3250 (90)	238 (40.9)	<0.001

COVID-19: corona-virus disease 2019; GI: gastrointestinal.

**Table 2:** Analysis of upper and lower GI endoscopy before and during the COVID-19 period.

Procedure Type/Result	Pre COVID-19 Time 3374	COVID-19 Time 457	*p*
**GI Endoscopy Indications, *n* (%)**			
Upper GI bleeding, *n* (%)	823 (24.4)	194 (42.5)	<0.001
Lower GI bleeding, *n* (%)	290 (8.6)	20 (4.4)	0.002
Anemia, *n* (%)	335 (9.9)	37 (8.1)	0.148
Chronic abdominal pain, *n* (%)	392 (11.6)	9 (2)	<0.001
Change in frequency of stool/vomiting, *n* (%)	245 (7.3)	34 (7.4)	0.895
Weight loss, *n* (%)	79 (2.3)	4 (0.9)	0.032
Control of gastric ulcer/polyps/cancer, *n* (%)	175 (5.2)	6 (1.3)	0.001
CT/MRI suspicion of neoplasia	30 (0.9)	0	0.042
Others (dysphagia, screening for esophageal varices), *n* (%)	1005 (29.8)	153 (33.5)	0.929
**GI Endoscopy Results**			
GI neoplasia, *n* (%)	249 (7.4)	57 (12.5)	0.001
GI polyps, *n* (%)	468 (13.9)	9 (2)	<0.001
Gastric/duodenal ulcer, *n* (%)			
Forrest 3, *n* (%)	185 (5.5)	40 (8.8)	0.002
Forrest 1-2, *n* (%)	242 (7.2)	45 (9.8)	0.69
Esophageal varix, *n* (%)	364 (10.8)	80 (17.5)	<0.001
Others (gastritis, esophagitis, diverticular disease, hemorrhoids), *n* (%)	1666 (46.4)	201 (44)	0.154
UC/Crohn’s disease, *n* (%)	91 (2.7)	18 (3.9)	0.134
Normal findings, *n* (%)	69 (2)	1 (0.2)	0.006
Achalasia, *n* (%)	35 (1)	3 (0.7)	0.616
Esophageal benign stenosis, *n* (%)	5 (0.1)	3 (0.7)	0.60
**Interventions Performed**			
Variceal band ligation, *n* (%)	42 (1.24)	14 (3.06)	0.715
Ulcer hemostasis, *n* (%)	97 (2.87)	21 (4.59)	0.039
Bougie dilation of esophageal benign strictures, *n* (%)	14 (0.41)	4 (0.87)	0.792
Balloon dilation of esophageal benign strictures, *n* (%)	24 (0.71)	7 (1.53)	0.987
Esophageal cancer stent placement, *n* (%)	5 (0.14)	1 (0.2)	0.610
Polypectomy/EMR, *n* (%)	170 (5.03)	3 (0.2)	<0.001
PEG, *n* (%)	5 (0.14)	2 (0.43)	0.752
Pyloric balloon dilation, *n* (%)	1 (0.02)	0	0.954
Success, *n* (%)	325 (90.7)	49 (94.2)	0.01
Early complications (bleeding/perforation), *n* (%)	69 (19.2)	4 (7.6)	<0.001
Delayed complications (bleeding/perforation), *n* (%)	7 (1.9)	0 (0)	0.395

GI: Gastrointestinal; ERCP: Endoscopic retrograde cholangiopancreatography; CT: Computed tomography; MRI: Magnetic resonance imaging; UC: Ulcerative colitis; EMR: Endoscopic mucosal resection; PEG: Percutaneous endoscopic gastrostomy.

**Table 3:** Analysis of ERCP before and during the COVID-19 period.

Procedure Type/Result	Pre COVID-19 Time 233	COVID-19 Time 124	*p*
**ERCP indications**			
Cholangitis, *n* (%)	25 (10.7)	16 (12.9)	0.540
CBD stones, *n* (%)	164 (70.4)	81 (65.3)	0.302
Cholangiocarcinoma, *n* (%)	32 (13.7)	21 (16.9)	0.700
Pancreatic cancer, *n* (%)	22 (9.4)	15 (12.1)	0.491
Postoperative biliary lesion, *n* (%)	6 (2.6)	2 (1.6)	0.171
Others (lymph node metastasis/hepatic metastasis)	7 (3)	4 (3.2)	0.852
**ERCP Results**			
Complications			
Immediate bleeding Perforation	27 (11.6) 1 (0.4)	9 (7.3) 1 (0.8)	0.196 0.649
Stent placement	50 (21.4)	30 (24.1)	0.378
Therapeutic success	199 (87.7)	90 (76.3)	0.006

ERCP: endoscopic retrograde cholangiopancreatography.

## Data Availability

The data presented are included in this study; additional data may be provided by the corresponding author on request.
